# HHLA2 is expressed in pancreatic and ampullary cancers and increased expression is associated with better post-surgical prognosis

**DOI:** 10.1038/s41416-020-0755-4

**Published:** 2020-02-19

**Authors:** Patrick P. C. Boor, Kostandinos Sideras, Katharina Biermann, M. Hosein Aziz, Iris J. M. Levink, Shanta Mancham, Nicole S. Erler, Xudong Tang, Casper H. van Eijck, Marco J. Bruno, Dave Sprengers, Xingxing Zang, Jaap Kwekkeboom

**Affiliations:** 1 Erasmus MC-University Medical Center, Department of Gastroenterology and Hepatology, Rotterdam, The Netherlands; 2 Erasmus MC-University Medical Center, Department of Surgery, Rotterdam, The Netherlands; 3 Erasmus MC-University Medical Center, Department of Pathology, Rotterdam, The Netherlands; 4 Erasmus MC-University Medical Center, Department of Biostatistics, Rotterdam, The Netherlands; 5 Department of Microbiology and Immunology, Albert Einstein College of Medicine, Bronx, NY USA

**Keywords:** Tumour biomarkers, Tumour immunology

## Abstract

**Background:**

HHLA2 is a recently discovered member of the B7-family of immune checkpoint molecules with limited expression in normal tissues but overexpression in several types of cancer. The aim was to determine the expression, prevalence and biological relevance of HHLA2 protein expression in two closely related human cancer types, namely pancreatic cancer and ampullary cancer.

**Methods:**

HHLA2 expression levels were retrospectively determined by immunohistochemistry in tissue micro-arrays of surgically resected tumours of 122 pancreatic cancer patients and 72 patients with ampullary cancer of the pancreato-biliary subtype.

**Results:**

HHLA2 was expressed at variable levels by tumour cells in 67% of pancreatic tumours and 93% of ampullary tumours. In the combined cohort high tumoural HHLA2 expression levels were significantly associated with delayed cancer recurrence and improved post-operative cancer-specific survival. The association of HHLA2 expression with cancer-specific survival and recurrence was statistically significant for the pancreatic cancer subgroup while a similar trend was found for the ampullary cancer subgroup. In multivariable analysis together with clinicopathologic characteristics, higher HHLA2 expression was an independent predictor of cancer-specific survival.

**Conclusion:**

The wide expression of HHLA2 in tumour cells and its association with cancer recurrence and patient survival suggest that HHLA2 represents a relevant immune checkpoint molecule in pancreatic and ampullary cancers.

## Background

Pancreatic cancer is one of the most fatal malignancies and a leading cause of cancer-related death in the world.^1^ Due to its late presentation, 5-year survival is a dismal 7%. Only 15–20% of patients are candidates for surgical resection with the 5-year survival, in these patients, only improving to 22%.^2^ Ampullary cancers arise from the ampullary of Vater. Whereas pancreatic cancer is worldwide the 14th most common type cancer, ampullary cancers are more rare.^1^ In western countries, only 16–35% of cancers resected by pancreatico-duodenectomy are ampullary cancers.^3^ Ampullary tumours are classified as intestinal or pancreato-biliary subtypes. Less than half of ampullary tumours belong to the pancreato-biliary subtype.^4^ They often grow into the pancreas and are histologically indistinguishable from pancreatic cancer, highly similar to pancreatic cancer at the molecular level, and are treated similarly to pancreatic cancer.^3,5,6^ However, patients with ampullary cancer generally present earlier in the disease course than patients with pancreatic cancer because of symptoms that result from biliary obstruction. As a result, surgical resection is possible in approximately half of ampullary cancer patients and, therefore, their prognosis is better than that of patients with pancreatic cancer. Nevertheless, the majority of patients eventually succumb to recurrent disease and die.^5^ Chemotherapy is minimally effective in both types of cancer.^7–9^ Thus, novel therapeutic strategies against pancreatic and ampullary cancers are needed.

Cancer immunotherapy is a rapidly developing type of cancer treatment, aiming to stimulate the immune system to combat and eliminate tumours. Among the various immunotherapeutic strategies, immune checkpoint inhibitors, such as antibodies against CTLA-4, PD-1, or its ligand PD-L1, have been proven successful in the treatment of several types of cancer.^10^ These immune checkpoint antibodies interrupt immune resistance mechanisms exploited by tumours to evade natural anti-tumour T-cell immunity. CTLA-4 and PD-1 are co-inhibitory receptors that are expressed on subsets of T cells. Ligation by their cognate ligands expressed on tumour cells and/or immune cells within the tumour microenvironment suppresses T-cell cytotoxicity against tumour cells. Unfortunately, both anti-PD-1 and anti-CTLA4 antibody therapy are not effective in pancreatic cancer,^11,12^ except in rare patients with mismatch repair deficient tumours,^13^ which account for less than 2% of pancreatic tumours.^13,14^ On the other hand, immune checkpoint antibody therapy has not been studied yet in ampullary cancer.

Therefore, it is relevant to investigate whether other immune checkpoint molecules might serve as targets for immunotherapy in pancreatic cancer. HHLA2, also known as B7H7, B7-H5 or B7y, is a recently discovered member of the B7 family of immune checkpoint molecules, which include B7–1 (CD80), B7–2 (CD86), B7-H1 (PD-L1), B7-DC (PD-L2), B7-H2 (ICOS-L), B7-H3, B7-H4 (B7x, B7S1, VTCN1), B7-H5 (VISTA), and B7-H6 (NCR3LG1). HHLA2 is not expressed in mice and its function is still unclear as both co-stimulatory and co-inhibitory effects of HHLA2 on human T cells have been described.^15–17^ Currently, the co-stimulatory molecule CD28H (also known as TMIGD2 or IGPR-1) is the only receptor that has been identified for HHLA2 to date, but the existence of a co-inhibitory receptor has been postulated.^18^ While HHLA2 is not expressed in most normal tissues, except for epithelial cells of gut, kidney, gall bladder and placenta,^19^ recent studies have reported expression of HHLA2 in tumour cells in several types of human cancers, such as breast cancer,^19^ osteosarcoma,^20^ lung cancer,^17,21^ colorectal carcinoma,^22^ bladder cancer^23^ and renal cell carcinoma.^24^ Whether HHLA2 is expressed in pancreatic cancer became recently a matter of controversy. Byers et al.^25^ reported HHLA2 expression in ductal cells in normal pancreas tissue but weak or no expression in pancreatic adenocarcinoma cells. On the contrary, Yan et al^26^ recently reported no or weak HHLA2-expression in normal pancreas tissue, but prevalent expression in pancreatic adenocarcinoma. Whether HHLA2 is expressed in ampullary cancer is as yet unknown.

The aim of the present retrospective study was to examine the expression of HHLA-2 in tumours of patients that underwent resection of pancreatic cancer or the pancreato-biliary subtype of ampullary cancer, and to study its biological relevance in these cancer types by relating its expression to baseline known clinicopathologic factors and outcome.

## Methods

### Patient population and tissue samples

This study was performed using tissue micro-arrays (TMA) containing five 1-mm cores from formalin fixed paraffin-embedded tumour tissues of 194 patients who underwent resection of pancreatic cancer (*n* = 122) or pancreato-biliary subtype of ampullary cancer (*n* = 72) at the Erasmus University Medical Center (Erasmus MC) between December of 2000 and June 2019. Scattered between these cores, we included cores of healthy placenta, kidney, testis, spleen, ovary and duodenum tissues. Further details on TMA construction can be found in a previous paper.^27^ Baseline clinicopathologic characteristics were retrospectively collected from electronic patient records. Follow-up information was updated till 31 December 2018. Median follow-up time was 19.1 months (range 0.1–128.2). Patients with duodenal, extrahepatic bile duct carcinomas, as well as all endocrine neoplasms were excluded. In addition, we excluded patients who died from post-operative complications. The study protocol was approved by the Medical Ethical Committee of Erasmus MC. Clinicopathologic information of the patients is shown in Table 1.

**Table 1:** Baseline characteristics.

Baseline characteristics		*N* = 194 (% or range)
Age^a^		67.9 (35.5–85.2)
Gender (male/female)		116 (59.8%)/78 (40.2%)
Positive margins^b^		71 (36.6%)
Lymph nodes metastasis		119 (61.3%)
CA-19.9 (kU/l)^a^		73.0 (1–6556)
Differentiation	Well-differentiated	17 (8.8%)
	Moderate	117 (60.3%)
	Poor	60 (30.9%)
Origin (pancreas/ampulla)		122 (62.9%)/72 (37.1%)
T-stage^c^ (pancreas/ampulla)	T1	20 (16.4%)/11 (15.3%)
	T2	85 (69.7%)/18 (25.0%)
	T3	17 (13.9%)/40 (55.6%)
	T4	0 (0%)/3 (4.2%)
Adjuvant chemotherapy		71 (36.6%)
Systemic chemotherapy^d^		92 (47.4%)
Peri-operative draining		141 (72.7%)
Surgical Procedure	Whipple	139 (71.6%)
	PPPD	40 (20.6%)
	Distal pancreatectomy	15 (7.7%)
Recurrence		140 (72.2%)
Cancer-specific death^f^		135 (69.6%)

^a^Median.

^b^Margins ≤ 1 mm included as positive.

^c^T-stage classification 8th edition (AJCC/UICC 2016).

^d^Any systemic adjuvant of palliative chemotherapy.

^e^Pylorus-sparing pancreatico-duodenectomy.

^f^8 patients died from causes other than pancreas or ampulary cancer (Cerebrovascular accident 3 × , myocardial infarction 2 × , multiple comorbidities 1 × , adjuvant chemo-radiation related 1 × , urothelial carcinoma 1 × ).

### HHLA2 immunohistochemistry

HHLA-2 was immunohistochemically stained using a specific mouse monoclonal antibody (clone 566.1) that was previously generated^15^ and validated for HHLA-2 immunohistochemistry in formalin fixed paraffin-embedded tissues.^19^ This antibody has since then been used for immunohistochemical studies on HHLA-2 expression in human breast cancer,^19^ lung cancer^17,21^ and osteosarcoma.^20^ TMA sections were deparaffinised followed by antigen retrieval in 10 mM sodium citrate buffer (pH 6.0) in a microwave for 10 minutes. Endogenous peroxidase activity was blocked in 0.3% H_2_O_2_ for 15 minutes. After goat serum block, 20 µg/ml of the primary antibody was applied at 4 °C overnight. HRP-conjugated goat anti-mouse IgG polymer secondary antibody (EnvisionTM, DAKO) was then applied for 1 h at room temperature, followed by DAB as chromogen. The slides were counter-stained with haematoxylin. Negative control stains consisted of omission of the primary antibody. In addition, the different healthy tissue cores in the TMA’s were used as positive and negative controls for HHLA2 staining. Staining intensity of HHLA2 on cancer cells was scored, for each core, as absent, low, intermediate or strong by two independent investigators (PPCB an KS) blinded to clinical outcome and differences were resolved by mutual agreement. In 20% of tumours HHLA2 expression levels differed between individual tumour tissue cores. In these cases, average scores from the 5 tumour cores were used for further analysis.

### HHLA2 mRNA quantification

HHLA2 mRNA expression was quantified by SYBR Green-based RT-PCR in frozen tumour tissues from 23 patients of our study cohort. Intron spanning primers were designed using the NCBI database. Forward primer: 5-CTTACCATCCACACAGTGC-3 and Reverse primer: 5-GCACCAAAATCCATAAGCC-3. HHLA2 mRNA levels were normalised to the geometric mean of GUSB, PMM1 and HPRT1 mRNA levels. The RT-PCR amplification technique was previously described.^28^

### Statistical analysis

Distributions of clinic-pathologic characteristics over HHLA-2 expression categories were examined using the Fischer’s exact test or the Kruskal-Wallis test as appropriate. For CA19–9 levels, we used the reference value as cut-off. Survival curves were estimated by the Kaplan-Meier method. Cancer-specific survival and recurrence-free survival were calculated from the date of surgery to the date of event (death from cancer or recurrence of cancer, respectively). In case of no event patients were censored at the date of last follow-up. The log-rank test was used to evaluate differences between survival curves of different groups. For multivariate analysis, the Cox proportional hazard regression analysis was used with backward variable selection. The statistical analysis was performed using the SPSS© 21 software.

## Results

### HHLA2 expression in pancreatic and ampullary cancer

**Fig. 1:**
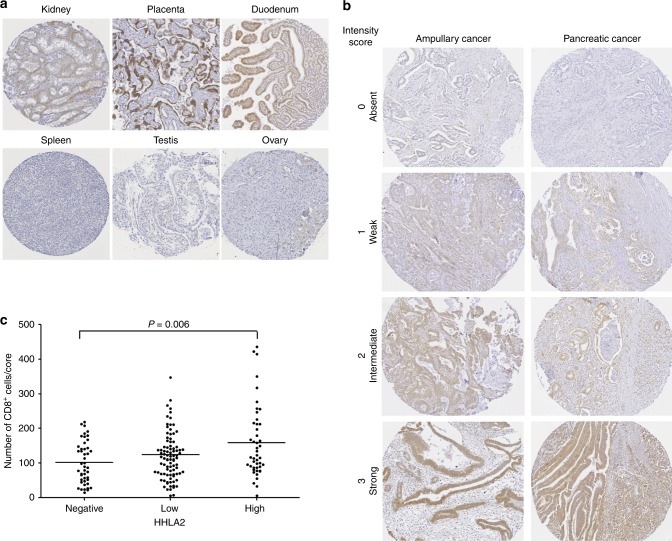
HHLA2 expression in pancreatic cancer and ampullary cancer of the pancreato-biliary subtype. **a** Immunohistochemical staining with anti-HHLA2 monoclonal antibody clone 566.1 shows HHLA2 expression on tubular epithelium of kidney, trophoblastic cells of placenta and duodenal epithelium, but no expression in healthy lung, spleen and ovary (magnification 28 × ). **b** Representative examples of the three different HHLA2 expression levels in ampullary cancer and pancreatic cancer (magnification 45 × ). **c** Numbers of tumour-infiltrating CD8^+^ T cells in tumours with different HHLA2 expression levels. *Y*-axis: Mean numbers of CD8^+^ T cells per core of tumour tissue, derived from CD8^+^ cell counts from five different tumour cores. Each case is depicted as a dot, and means are shown as horizontal lines.

Specificity of HHLA2 immunohistochemistry was investigated by evaluation of the staining of cores of different healthy human tissues in the TMA’s. We observed HHLA2 expression on tubular epithelium of kidney, trophoblastic cells of placenta and duodenal epithelium, but no expression in spleen, testis and ovary (Fig. 1a), which is in accordance with published HHLA2 immunohistochemistry data.^19^

Because the pancreato-biliary subtype of ampullary cancer and pancreas cancer are closely related cancer types, we first analysed HHLA2 expression in the combined cohort. Consistent with previous reports on HHLA2 expression in several types of cancer, including pancreatic cancer,^19,21,22,26^ we observed both membranous and cytoplasmic expression in cancer cells. HHLA2 staining intensity of cancer cells was graded as absent, low, intermediate or strong (Fig. 1b). Due to the limited number of cancer cells that were graded as strong, the strong and intermediate groups were combined to one group that we labelled as high HHLA-2 expressers. In 77% of tumours we observed HHLA2 expression in cancer cells; expression was graded as low in 49% and as high in 28% of tumours. In addition, expression of HHLA2 mRNA was detected in 23 out of 23 tested tumour tissues and, similar to protein expression, was strongly variable between individual patients. HHLA2 mRNA levels in tumours were not associated to protein expression levels as determined by immunohistochemistry (Supplementary Fig. 1). This discrepancy may partially be caused by the fact that we focused on cancer cell expression while HHLA2 is also expressed in the tumour microenvironment such as immune cells, stromal cells and neural tissue (Fig. 1b and Supplementary Fig. 2). The expression of HHLA2 on non-cancerous cells was not scored. This is also in concurrence with the opinion that there is no strong association between mRNA level and protein expression of B7-H3 or B7x.^29^

Analysis of associations of HHLA-2 expression with clinicopathologic characteristics (Table 2) showed that HHLA2 was more frequently expressed in ampullary tumours compared to pancreatic tumours (93% vs 67%). Moreover, 53% of ampullary tumours expressed high levels of HHLA2 compared to 13% of pancreatic tumours. Especially few T2 stage pancreatic tumours showed high HHLA-2 expression. The distribution of CA-19.9 levels differed across HHLA2 expression levels. Closer inspection of the median CA-19.9 levels within each HHLA2 group indicated that there may be an inverse relation between CA-19.9 and HHLA2 levels.

**Table 2:** Baseline characteristics.

Baseline characteristics		HHLA2 absent (*n* = 45)	HHLA2 Low (*n* = 95)	HHLA2 High (*n* = 54)	*P* Value
Age in years^a^		65.1 (35.5–82.1)	69.6 (43.8–85.2)	66.9 (37.6–83.0	0.398
Gender (male/female)		23 (19.8%)/22 (28.2%)	60 (51.7%)/35 (44.9%)	33 (28.4%)/21 (26.9%)	0.401
Positive margins		20 (28.2%)	40 (56.3%)	11 (15.5%)	0.012
Lymph nodes metastasis		31 (26.1%)	60 (50.4%)	28 (23.5%)	0.193
CA-19.9 (kU/l)^a^		143.0 (1–3440)	70.0 (1–6556)	38.0 (1–718)	0.000
Differentiation	Good	4 (23.5%)	8 (47.1%)	5 (29.4%)	
	Moderate	25 (21.4%)	54 (46.2%)	38 (32.5%)	0.377
	Poor	16 (26.7%)	33 (55.0%)	11 (18.3%)	
Origin (pancreas/ampulla)		40 (32.8%)/ 5 (6.9%)	66 (54.1%)/29 (40.3%)	16 (13.1%)/38 (52.8%)	0.000
T-stage^b^ (pancreas/ampulla)	T1	5 (25.0%)/ 0 (0%)	9 (45.0%)/4 (36.4%)	6 (30.0%)/7 (63.6%)	
	T2	29 (34.1%)/ 1 (5.6%)	50 (58.8%)/6 (33.3%)	6 (7.1%)/11 (61.1%)	0.042/0.855
	T3	6 (35.3%)/ 4 (10.0%)	7 (41.2%)/17 (42.5%)	4 (23.5%)/19(47.5%)	
	T4	0 (0%)/ 0 (0%)	0 (0%)/2 (66.7%)	0 (0%)/1 (33.3%)	
Peri-operative draining		35 (24.8%)	64 (45.4%)	42 (29.8%)	0.288
Surgical procedure	Whipple	34 (24.5%)	70 (50.4%)	35 (25.1%)	
	PPPD	7 (17.5%)	16 (40.0%)	17 (42.5%)	0.191
	Distal pancreatectomy	4 (26.7%)	9 (60.0%)	2 (13.3%)	
Adjuvant chemotherapy		23 (32.4%)	33 (46.5%)	15 (21.1%)	0.052
Systemic chemotherapy		27 (29.3%)	46 (50.0%)	19 (20.7%)	0.029

^a^Median. For patients that underwent peri-operative draining, we used post-drainage CA19-9 values.

^b^8th edition of T-stage classification.

In our previous study,^27^ we counted numbers of tumour-infiltrating CD8^+^ cytotoxic T cells and Foxp3^+^ regulatory T cells per tumour core in the same TMA as we used in the current study. Here, we analysed whether HHLA2 expression levels correlated with numbers of tumour-infiltrating CD8^+^ or Foxp3^+^ T cells. Tumours with high HHLA2 expression levels contained slightly, but significantly, more tumour-infiltrating CD8^+^ cells compared to tumours without HHLA2 expression (Fig. 1c). HHLA2 expression was not associated with numbers of infiltrating Foxp3^+^ cells (data not shown).

### HHLA2 expression is associated with better post-surgical prognosis

**Fig. 2:**
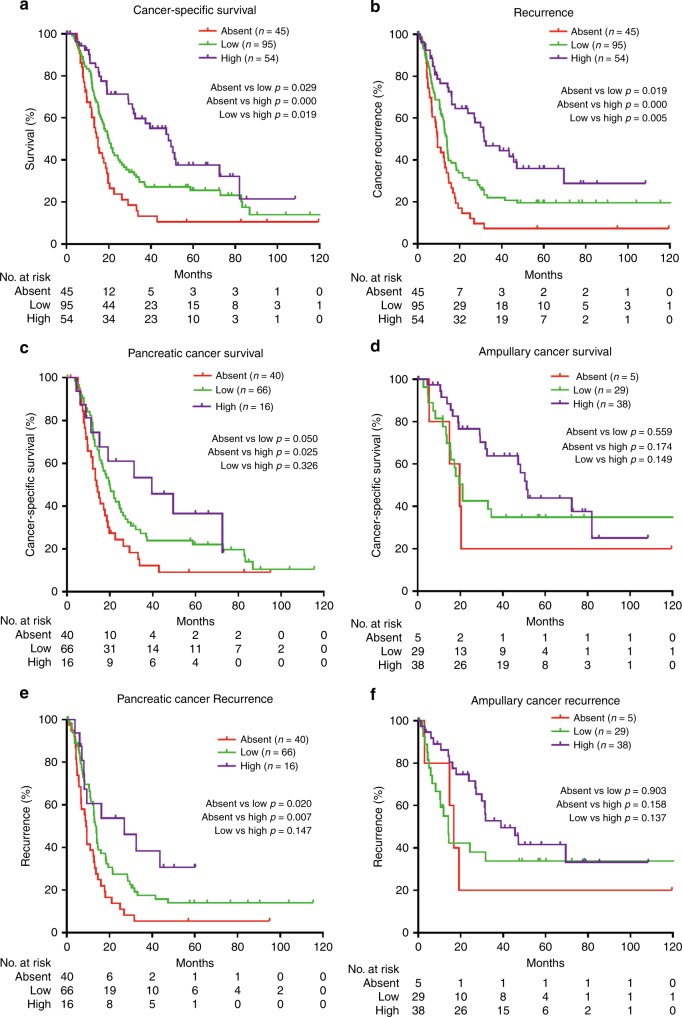
Associations of HHLA2 expression in tumours with patient survival and tumour recurrence. **a** Kaplan-Meier plot showing post-resection survival time of patients with pancreatic cancer or ampullary cancer with no, low or high HHLA2 expression in their tumours. **b** Kaplan-Meier plot showing recurrence-free survival after tumour resection of patients with pancreatic cancer or ampullary cancer with no, low or high HHLA2 expression in their tumours. **c** Kaplan–Meier plot showing post-resection survival time of pancreatic cancer patients with no, low or high HHLA2 expression in their tumours. **d** Kaplan–Meier plot showing post-resection survival time of ampullary cancer patients with no, low, or high HHLA2 expression in their tumours. **e** Kaplan–Meier plot showing recurrence-free survival after tumour resection of patients with pancreatic cancer with no, low or high HHLA2 expression in their tumours. **f** Kaplan–Meier plot showing recurrence-free survival after tumour resection of patients with ampullary cancer with no, low or high HHLA2 expression in their tumours.

Higher HHLA2 expression in cancer cells was significantly associated with improved post-operative cancer-specific survival. Patients with high HHLA2 expression showed the best survival, while patients without HHLA2 expression showed the worst survival (Fig. 2a). Likewise, increased HHLA2 expression in cancer cells was positively associated with delayed cancer recurrence (Fig. 2b). Intratumoural HHLA2 expression was also positively associated with survival and recurrence in pancreatic cancer patients alone (Fig. 2c, e), while in ampullary cancer patients a similar trend was observed, but the differences were not statistically significant (Fig. 2d, f).

In univariate analysis, HHLA2 expression, T-stage, tumour differentiation grade, margin status, lymph node status, CA-19.9 level and tumour location (ampulla versus pancreas) were significantly associated with cancer-specific survival (Table 3). In multivariable analysis, HHLA2 expression, tumour differentiation grade (poor as compared to well-differentiated), margin status and lymph node status were independent predictors of cancer-specific survival (Table 3). This analysis indicates that HHLA2 expression in tumour cells is, independent of clinical or pathological characteristics, predictive for survival after tumour resection in a combined cohort of pancreatic and ampullary cancer patients. Importantly, tumour location was not an independent predictor of cancer-specific survival when corrected for clinicopathologic factors.

**Table 3:** Univariable and multivariable Cox proportional hazard regression analysis of patients cancer-specific survival.

Variables	Univariable Analysis		Multivariable analysis	
	*P*	HR (95% CI)	*P*	HR (95% CI)
HHLA2-expression				
Absent vs low	0.024	0.629 (0.421–0.940)	0.022	0.601 (0.389–0.929)
Absent vs high	0.000	0.371 (0.227–0.607)	0.011	0.471 (0.264–0.839)
T-stage (T1 vs T2, T3, T4)	0.012	1.992 (1.163–3.411)	0.555	1.187 (0.672–2.096)
Tumour differentiation				
Poor vs moderate	0.784	0.915 (0.486–1.724)	0.666	0.855 (0.419–1.742)
Poor vs well-differentiated	0.046	1.946 (1.010–3.761)	0.005	0.561 (0.377–0.836)
Margin Status	0.000	2.212 (1.564–3.127)	0.000	2.003 (1.360–2.950)
Lymph node status	0.000	2.844 (1.946–4.157)	0.000	2.734 (1.797–4.160)
Ca19-9 (>37 vs<37 kU/L)	0.001	1.940 (1.321–2.850)	0.297	1.264 (0.814–1.962)
Pancreas vs Ampulla	0.001	1.873 (1.292–2.715)	0.623	1.119 (0.715–1.750)
Systemic chemotherapy	0.058	1.408 (0.989–2.005)	0.600	1.110 (0.751–1.642)
Age, yrs	0.095	0.985 (0.967–1.003)	0.310	0.990 (0.970–1.010)

## Discussion

This study is the first to show that HHLA2 is widely expressed in tumour cells of patients with ampullary cancer of the pancreato-biliary subtype. In pancreatic cancer HHLA2 expression has been investigated in two previous studies.^25,26^ Our data are similar to those of Yan et al.^26^ by showing that HHLA2 is expressed in tumour cells of the majority (67%) of pancreatic cancer patients. Yan et al. reported expression in 77% of pancreatic tumours. We used a different anti-HHLA2 antibody clone for immunohistochemistry than Yan et al. Therefore, the similarity of expression data in both studies can be regarded as strong evidence that HHLA2 protein is expressed in the majority of pancreatic cancers. In addition, we demonstrate that HHLA2 mRNA was expressed, although at variable levels, in all pancreatic and ampullary cancers examined. In contrast, Byers et al.^25^ reported that HHLA2 expression is absent or at most weak in pancreatic tumour cells. This report is difficult to reconcile with our findings and those of Yan et al. due to the lack of information on the primary antibody utilised by Byers et al.

Similar to Yan et al.,^26^ we found that increased intratumoural HHLA2 expression is associated with improved patient survival after tumour resection in pancreatic cancer patients. Compared to the study of Yan et al.,^26^ our study has several improvements. First, we demonstrate that higher HHLA2 expression is not only associated with prolonged survival, but also with delayed cancer recurrence. Moreover, by comparing three HHLA2 expression level categories (absent, low, high expression) instead of two categories, we show that increases in HHLA2 expression are associated with prolonged time to recurrence and cancer-specific death in a dose-dependent fashion. Finally, by multivariable analysis we demonstrate that increased HHLA2 expression levels are associated with better survival, independently of clinicopathologic characteristics, while such analysis is absent in the report of Yan et al.

In ampullary cancer, we found similar trends as in pancreatic cancer patients, but associations of HHLA-2 expression with recurrence or survival did not reach statistical significance. This is probably caused by the limited size of our ampullary cancer cohort compared to the pancreatic cancer cohort (*n* = 72 versus *n* = 122). Ampullary cancer is a rare cancer type, and we included all tumour tissues resected between December 2000 and January 2019 that were archived in our centre and could unfortunately not enlarge our cohort further. Therefore, our data on the biological significance of HHLA-2 expression in ampullary cancer require validation in a larger, probably multi-centre cohort of this type of cancer.

In contrast to our observations and those of Yan et al. in pancreatic and ampullary cancers, intratumoural HHLA2 expression levels were associated with increased risk of cancer recurrence or decreased survival in triple-negative breast cancer, renal cell carcinoma osteosarcoma, colorectal cancer and bladder urothelial carcinoma.^19,20,22–24^ One reason for these contrasting clinical associations may be related to different methods of scoring HHLA-2 expression. While in our study as well as in the study of Yan et al., HHLA2 expression in tumour cells was scored while expression on stromal cells was neglected, several other studies might have scored expression on both tumour and stromal cells.^22–24^ In addition, the contrasting clinical associations may be related to different tumour microenvironments or opposite immunological functions of HHLA2 in different cancer types, since both co-stimulatory and co-inhibitory effects of HHLA2 on human T cells have been described.^15–17^ Therefore, the existence of an as yet unidentified co-inhibitory receptor for HHLA2 in addition to the known co-stimulatory receptor CD28H has been postulated.^18^ Similarly contrasting results between protein expression and patient outcome have also been reported for different types of cancer for the well-studied co-inhibitory B7 member PD-L1.^27,28,30–33^ While the association of increased intratumoural expression of the co-inhibitory PD-L1 molecule with reduced cancer recurrence and/or improved patient survival is at first instance counterintuitive, it has been explained by the phenomenon of adaptive immune resistance. This means that tumour cells up-regulate expression of inhibitory immune checkpoint molecules in response to T-cell infiltration to evade immune attack.^32,34^ Mechanistically, this phenomenon is caused by induction of PD-L1 expression on tumour cells by cytokines such as IFN-γ that are secreted by tumour-infiltrating T cells.^35^ Our observation that tumours with high HHLA2 expression contained (slightly) higher numbers of tumour-infiltrating CD8^+^ T cells may favour the hypothesis that HHLA2 expression in pancreatic and ampullary tumour cells can also be induced by infiltrating T cells, and in fact may be regarded as a sign of active immune attack. This hypothesis may explain the observed association of increased HHLA2 expression with improved survival. Unfortunately, no data on the regulation of HHLA2 expression in tumour cells by immunological mechanisms are as yet available. Interestingly, Yan et al^26^ reported HHLA2 expression in inflamed ducts in peritumoural tissues in 10% of pancreatic cancer patients, while normal duct epithelial cells do not express HHLA-2, suggesting that inflammation can induce HHLA-2 expression on pancreatic ductal epithelial cells. Moreover, they also observed HHLA-2 expression in precancerous pancreas lesions present in tumour-surrounding tissues, suggesting that induction of HHLA2 in pancreatic cancer cells may already occur early during oncogenesis. Unfortunately, we had limited potential to systemically examine HHLA2 expression in peritumoural tissues.

While pancreatic cancer and ampullary cancer of the pancreato-biliary subtype are histologically indistinguishable and show many similarities at the molecular level, we found that HHLA2 was more frequently expressed in ampullary tumours compared to pancreatic tumours. Previously, we have studied PD-L1 expression in the same cohort as used in the current study.^27^ We now used these data to compare PD-L1 expression between both types of cancer and found that 85% of ampullary cancer patients showed PD-L1 expression in their tumours versus 71% of pancreatic cancer patients (*p* = 0.043). These data indicate that ampullary tumours may differ from pancreatic tumours in terms of higher expression of these two co-inhibitory molecules and suggest that ampullary cancer might be more sensitive for therapeutic blockade of these co-inhibitory pathways than pancreatic cancer.

In conclusion, HHLA2 is widely expressed in tumour cells of pancreatic cancer and ampullary cancers. Its association with cancer recurrence and patient survival suggest that HHLA2 may represent a biologically relevant immune checkpoint molecule in these cancer types and should be evaluated as a potential target for immunotherapy in pancreatic and ampullary cancers.

## Supplementary information

supplementary figures

## Data Availability

The datasets used and/or analysed during the current study are available from the corresponding author on reasonable request.
