# Supplementary material for: HHLA2 is expressed in pancreatic and ampullary cancers and increased expression is associated with better post-surgical prognosis
Source: Br J Cancer. 2020 Feb 19;122(8):1211–8. doi: 10.1038/s41416-020-0755-4 (PMC7156757; doi:10.1038/s41416-020-0755-4)
Supplement: Supplementary file 1 — supplementary figures [file 41416_2020_755_MOESM1_ESM.pptx]

## Slide 1
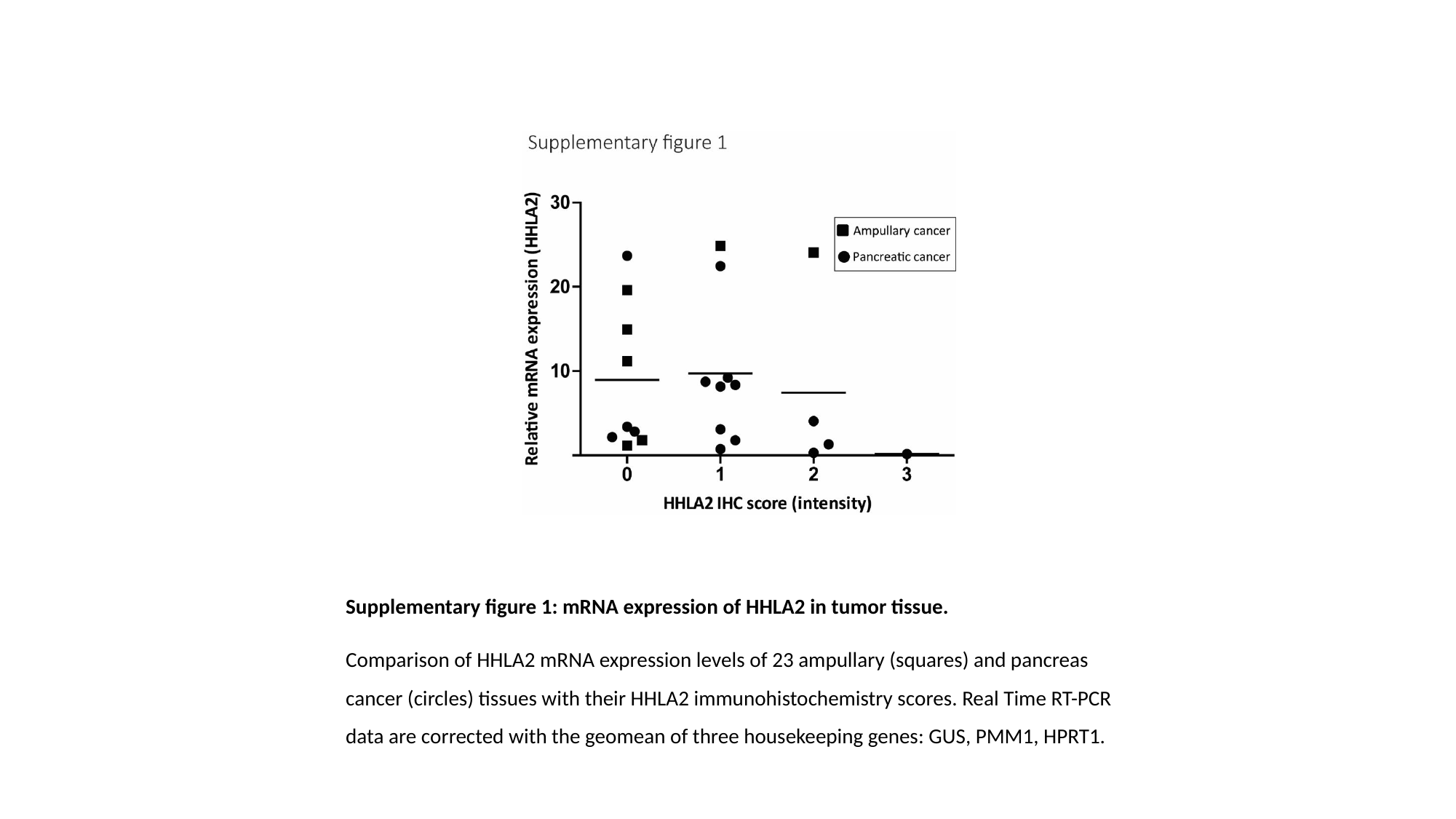

Supplementary figure 1: mRNA expression of HHLA2 in tumor tissue.
Comparison of HHLA2 mRNA expression levels of 23 ampullary (squares) and pancreas cancer (circles) tissues with their HHLA2 immunohistochemistry scores. Real Time RT-PCR data are corrected with the geomean of three housekeeping genes: GUS, PMM1, HPRT1.

## Slide 2
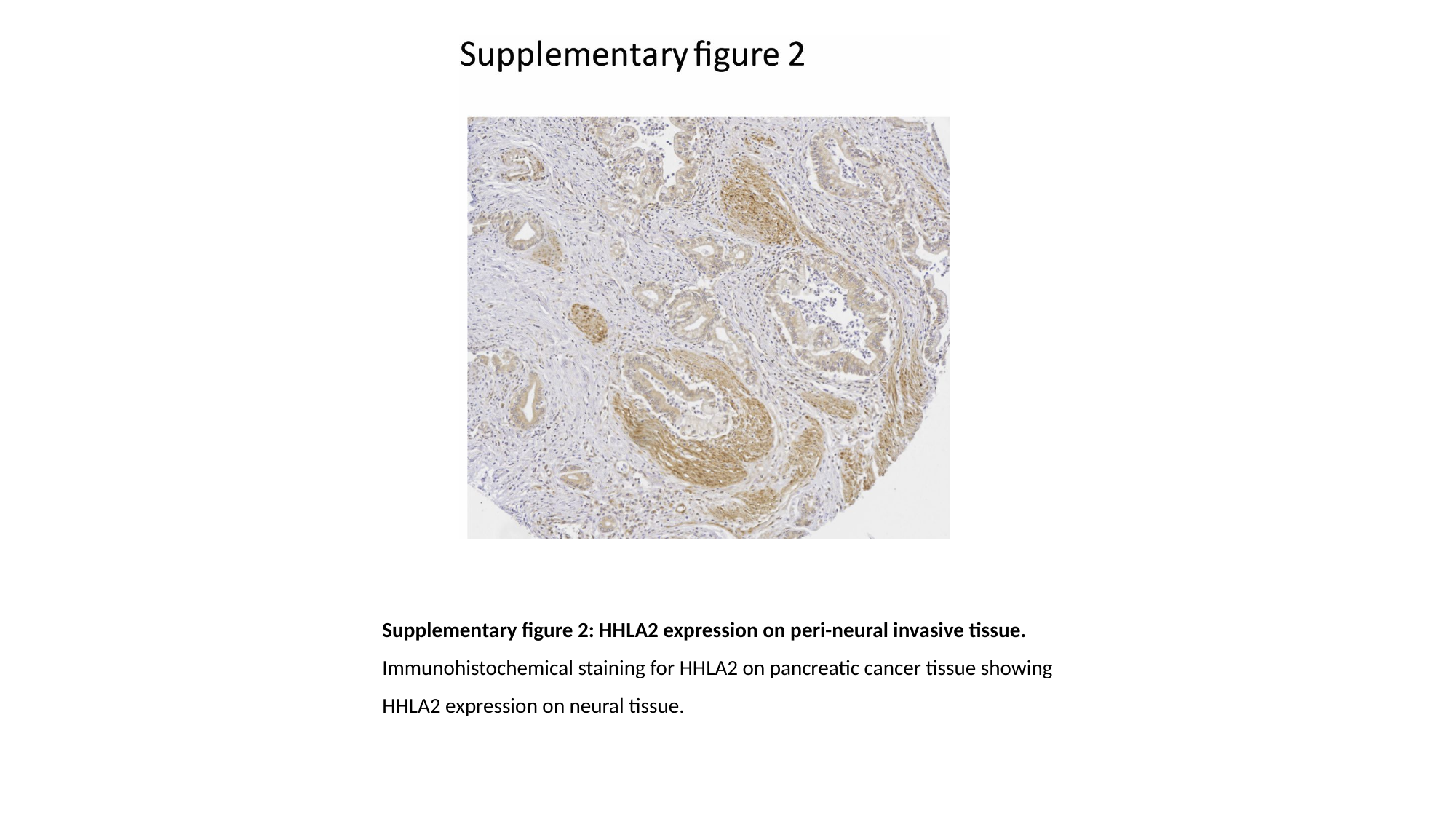

Supplementary figure 2: HHLA2 expression on peri-neural invasive tissue. Immunohistochemical staining for HHLA2 on pancreatic cancer tissue showing HHLA2 expression on neural tissue.
